# The Effect of Reflexology on Anxiety in Patients Undergoing Open Heart Surgery: Systematic Review and Meta-Analysis of Randomized Controlled Trials

**DOI:** 10.1097/jnr.0000000000000725

**Published:** 2026-01-26

**Authors:** Necibe DAĞCAN ŞAHİN, Selçuk GÖRÜCÜ

**Affiliations:** 1 Nursing Department, Faculty of Health Sciences, Kutahya Health Sciences University, Kutahya, Turkey; 2 Nursing Department, Faculty of Health Science, Akdeniz University Kumluca, Antalya, Turkey

**Keywords:** anxiety, open-heart surgery, reflexology, systematic reviews, nursing

## Abstract

**Background:**

Open-heart surgery is effective but may involve significant postoperative complications and long recovery times that can be exacerbated by patient anxiety. Nonpharmacological approaches such as reflexology have gained attention in the field of medicine for their ability to alleviate anxiety without significant side effects.

**Purpose:**

This systematic review and meta-analysis were designed to determine the effect of reflexology on anxiety in patients undergoing open-heart surgery.

**Methods:**

The PubMed, EBSCO (including MEDLINE and CINAHL), OVID, Web of Science, and Cochrane databases were searched from May 15 to July 15, 2024. Randomized controlled trials that evaluated patients who had open-heart surgery were included in the analysis. Studies were appraised using the Critical Appraisal Checklists for Randomized Controlled Trials developed by the Joanna Briggs Institute (JBI). The meta-analysis was conducted using the Comprehensive Meta-Analysis Version 2, and heterogeneity between studies was assessed using the χ^2^ test and *I* ^2^ statistic. The results were reported in line with the Preferred Reporting Items for Systematic Reviews and Meta-Analyses statement.

**Results:**

Eight studies encompassing 485 patients were included in the meta-analysis. All of the included studies used common measurement times for anxiety and compared intervention groups with either standard care or a placebo. In the meta-analysis performed using a random-effects model, reflexology was found to significantly reduce anxiety (*g*=−1.49, 95% confidence interval (CI)=[−2.35, −0.63], with a statistically significant difference observed between the intervention and control groups (*z*=−3.41, *p*=.001). However, the findings of the meta-analysis should be interpreted with caution.

**Conclusions:**

Reflexology may be effective in reducing anxiety in patients undergoing open-heart surgery. However, the significant heterogeneity identified among the included studies indicates variability among study conditions. Therefore, their outcomes should be evaluated carefully. Reflexology may be used as an adjunct to standard patient care. Additional studies with more homogeneous methodologies are required to strengthen the evidence base for the efficacy of reflexology in reducing anxiety in this vulnerable patient population.

## Introduction

Cardiovascular diseases are a leading cause of mortality worldwide (Göktuna et al., 2023). Coronary artery bypass graft (CABG) surgery, valve repair, valve replacement, and minimally invasive procedures such as coronary angiography, percutaneous transluminal coronary angioplasty, and percutaneous coronary intervention are commonly used in the treatment of coronary artery disease and heart valve disorders (Chandrababu et al., 2019; Soh et al., 2024). CABG and heart valve surgeries, which require open-heart procedures, are effective and often preferred in advanced cases (Soh et al., 2024). These surgeries are traditionally performed via median sternotomy, involving large and multiple incisions (Ünver et al., 2023). However, they also carry risks of complications that can delay healing (Soh et al., 2024).

Open-heart surgery is a highly stressful experience that significantly increases anxiety levels in patients (Chandrababu et al., 2023). Preoperative anxiety stems from uncertainty about surgical outcomes, while postoperative anxiety in intensive care and wards is triggered by factors such as insomnia, noise, pain, and inactivity (Kakar et al., 2021; Ramesh et al., 2017). In addition, postoperative complications and fear of death further contribute to anxiety (Chandrababu et al., 2023). Anxiety related to cardiac procedures affects body hemodynamics by altering sympathetic, parasympathetic, and endocrine system activity (Micheluzzi et al., 2024), and may increase the use of benzodiazepines and opioids, leading to prolonged hospital stays (Kakar et al., 2021). However, these pharmacological agents can cause respiratory and cardiac depression, nausea, dizziness, and extended recovery times (Whitehead et al., 2020). Furthermore, postsurgical anxiety and stress negatively impact recovery and the ability to cope in patients (Chandrababu et al., 2023; Ramesh et al., 2017).

Nonpharmacological and complementary treatments are increasingly preferred for use in interventions designed to enhance patients’ emotional, mental, and physiological health and reduce anxiety (Embong et al., 2016). These treatments are simple, cost-effective, noninvasive, and free of side effects (Chandrababu et al., 2019). One of these, reflexology, is a manual therapy that involves applying pressure to “reflex areas” on the feet, hands, and ears, influencing the health of corresponding body parts (Embong et al., 2016; Göktuna et al., 2023). First described in the literature by Dr. William Fitzgerald in the early 20th century, reflexology has two internationally accepted methods: Ingham and Rwo Shur. The Ingham method applies pressure manually, whereas the Rwo Shur method uses tools such as wooden sticks (Embong et al., 2016; Whatley et al., 2022). Although practiced in China and Egypt for centuries, reflexology has been used in the West for approximately 90 years (Öztürk et al., 2018). Due to its positive effects, reflexology has been increasingly studied in scientific research (Göktuna et al., 2023) and has been reported to improve blood flow and relieve tension (Embong et al., 2016). Moreover, massage therapy activates mechanoreceptors in the hands and feet, preventing pain signals from reaching higher processing centers, thus reducing anxiety levels (Sözen & Karabulut, 2020).

Reflexology involves applying deep pressure to the hands and feet, stimulating sensory receptors connected to specific body regions. This process enhances blood circulation and promotes relaxation by restoring energy balance (Ali et al., 2023; Göktuna et al., 2023). Similarly, massage therapy activates mechanoreceptors in the hands and feet, blocking pain signals from reaching higher processing centers. As pain decreases, anxiety levels may also be reduced (Sözen & Karabulut, 2020). In addition, individuals with anxiety tend to exhibit increased sympathetic activity and decreased parasympathetic responses. Reflexology reduces stress responses and increases relaxation effects by calming the body, relieving anxiety and helping reduce muscle tension (Brand et al., 2013).

Also, reflexology has been shown to strengthen the nurse-patient relationship (Chandrababu et al., 2019). Studies conducted on different samples have also reported reflexology as effective in reducing anxiety (Öztürk et al., 2018; Vahedparast et al., 2024). Hand and foot reflexology applied to patients undergoing cardiovascular procedures has been reported to have statistically significant, positive effects on anxiety (Göktuna et al., 2023; Gunes et al., 2024; Heidari et al., 2017; Mobini-Bidgoli et al., 2017; Shafiei & Akbari, 2017).

### The Significance of This Meta-Analysis

Few comprehensive reviews and meta-analyses in the literature address the impact of reflexology on anxiety in patients undergoing cardiovascular treatments (Chandrababu et al., 2019). All cardiovascular surgical procedures were considered in this systematic review and meta-analysis. Open-heart surgery, a more significant procedure than cardiovascular surgery procedures, requires a deeper incision due to the opening of the sternum. Therefore, evidence from previous studies may not be sufficient or discriminatory for patients undergoing open-heart surgery. Therefore, this systematic review and meta-analysis were conducted to examine the effect of reflexology on anxiety in patients undergoing open-heart surgery and provide new related evidence.

### Research Questions

The question addressed in this research is: What is the effect of reflexology on anxiety in patients undergoing open-heart surgery?

### Aim

This systematic review and meta-analysis were designed to determine the effect of reflexology on anxiety in patients undergoing open-heart surgery.

## Methods

### Design

This systematic review and meta-analysis were conducted and reported in accordance with the Preferred Reporting Items for Systematic Review and Meta-Analysis Statement-PRISMA (Page et al., 2021). The research protocol followed was documented in the PROSPERO database (ID: CRD42024573824). To minimize risk of bias, literature search, article selection, data extraction, and quality assessment procedures were conducted separately by two researchers. Before beginning the study, both researchers reviewed each stage of the process, and decisions were made through mutual discussion. A pilot study, including searching the PubMed database using the selected keywords, vetting articles, extracting data from the five selected articles, and assessing article quality, was conducted during joint working sessions with both researchers. These disagreements after the pilot study were resolved by consensus.

### Search Strategy

The search approach was developed using keywords associated with the PICO framework (patient or population, intervention, control or comparator, and outcomes) to identify pertinent studies. Original research published over the past two decades (2001–2024) examining the application of reflexology to reduce anxiety in patients undergoing cardiac surgery was reviewed. No previous studies were found in this area. Alternative sources, including PubMed, EBSCO (MEDLINE, CINAHL), OVID, Web of Science, and Cochrane databases, were utilized for searches. The following Medical Subject Headings (MeSH) terms and keyword groups were used: (“cardiac” OR “cardiac surgery” OR “cardiac surgery” OR “open heart” OR “open heart surgery” OR “coronary artery bypass” OR “coronary artery bypass graft” OR “CABG” OR “coronary artery bypass graft surgery” OR “sternotomy” OR “cardiovascular surgical procedures”) AND (“reflexotherapy” OR “reflexology”) AND (“anxiety”). The bibliographies of the studies incorporated in this research, along with prior compilations on the topic, were reviewed for further research.

### Study Selection

For this systematic review, randomized controlled trials (RCTs) designed to investigate and document the impact of reflexology on anxiety in patients undergoing coronary artery bypass grafting and open-heart surgery were chosen. The first and second researchers independently identified and selected studies based on the established inclusion and exclusion criteria. After eliminating duplicates during the screening process, the remaining articles were vetted in sequence by reviewing titles, abstracts, and full texts. The included studies involved adult patients (over 18 years old) undergoing coronary artery bypass grafting and open-heart surgery, and patients randomly assigned to either a treatment group receiving reflexology or a control group receiving standard care (e.g., clinic-administered monitoring of daily vital signs and balance, administration of physician-prescribed medications, and applying a light caressing massage to benefit from the placebo effect).

#### Eligibility Criteria

The articles included in this systematic review were vetted based on the PICOS criteria outlined below:

P: Patient: Adults undergoing open-heart surgery and undergoing coronary artery bypass grafting

I: Intervention: Foot, hand, or ear reflexology

C: Comparison: Usual or standard care or a placebo intervention

O: Outcomes: Primary outcome—results of anxiety assessment (as outlined in research studies).

S: Study design: Randomized controlled trials published in English.

#### Inclusion Criteria

The inclusion criteria were the following:

1. Type of study: (a) Original research articles published in English from 2001 to 2024; (b) RCTs designed to evaluate patients who had received CABG or valve surgery as a result of open-heart surgery.
2. Type of intervention: Effect of reflexology massage on postsurgery anxiety.
3. Participant type: Adult patients undergoing open-heart procedures such as CABG surgery and valve replacement or repair.

In this study, the primary outcome was the postoperative anxiety level following open-heart surgery or coronary artery bypass grafting. No secondary outcomes were assessed or evaluated. Studies employing unclear methodologies, providing incomplete data, or lacking accessible full texts were excluded from the analysis.

### Coding of the Studies

To gather information for this study, a data extraction tool created by the researchers based on earlier studies was utilized (Ramesh et al., 2017; Soh et al., 2024). Using this data extraction tool, information on the author, publication year, country, study design, the clinic where the study was performed, participant characteristics, sample size, intervention details, data collection methods, comparisons, and outcome measures was collected (Table 1).

**Table 1:** Summary of Studies, Interventions, Outcome Measures, and Findings

References/ Country	Study Design	Study Setting	Sample Size	Data Collection	Intervention and Features	Main Results
Abbaszadeh et al. (2018)/Iran	Randomized controlled trial (RCT)	Cardiac Surgery Unit of Shahid Madani Teaching Hospital	*N*=120 patients who underwent CABG surgery • Exp. group A: *n*=40 • Cont. group C: *n*=40	State-Trait Anxiety Inventory (STAI)	Group A received foot reflexology in bed while reclining in a supine position. The foot reflexology and surface heel massage of each foot lasted for 15 min. ❖ Foot reflexology methodology	Postintervention Anxiety levels Exp. group A: 6.21±0.82 Cont. group C: 7.80±2.31
Bagheri-Nesami et al. (2014)/Iran	RCT	Mazandaran Heart Centre	*N*=80 patients • Exp. group: *n*=40 • Cont. group: *n*=40	STAI	Lubricating cream was gently applied on the participants’ left feet for 1 min, followed by foot reflexology massage on the sole of the foot for 20 min. ❖ Foot reflexology massage	Postintervention Anxiety levels Exp. group: 9.70±3.75 Cont. group: 11.43±4.51
Göktuna et al. (2023)/Turkey	RCT	Cardiovascular surgery intensive care unit of a tertiary hospital	*N*=48 patients who underwent CABG surgery • Exp. group: *n*=24 • Placebo. group: *n*=24	STAI	This study has compared the effects of a 20-min hand-reflexology massage to a placebo stroking massage on pain and anxiety in newly extubated patients in the intensive care unit who had undergone CABG surgery. ❖ Hand-reflexology massage	Postintervention Anxiety levels Exp. group: 25.25±5.84 Placebo. group: 41.04±9.56
Gunes et al. (2024)/Turkey	RCT	Cardiovascular Surgery Service of a university health practice and research center hospital	*N*=70 patients who underwent CABG surgery • Exp. group: *n*=35 • Cont. group: *n*=35	STAI	The researcher applied foot reflexology to each patient under the supervision of a physician during the first, second, and third postoperative days. On average, each session of reflexology practice took 40 min per patient. ❖ Foot reflexology intervention	Postintervention Anxiety levels Exp. group: 23.69±2.9 Cont. group: 47.26±4.20
Gunnarsdottir & Jonsdottir, (2007)/Iceland	RCT	Landspitali University Hospital	Conducted on 9 patients undergoing CABG hospitalized • Exp. group: *n*=5 • Cont. group: *n*=4	STAI	After applying the feet with a neutral lubricant cream for 1 min, patients in the experimental group received reflexology for 30 minutes on both feet while lying supine in bed. ❖ Foot reflexology intervention	Postintervention Anxiety levels Exp. group: 1.36±5.8 Cont. group: 0.85 ±4.8
Kolbadinejad et al. (2023)/Iran	RCT	Intensive Care Unit of Fatemeh Zahra Hospital	Conducted on 66 patients undergoing CABG hospitalized • Exp. group: *n*=28 • Cont. group: *n*=28	The Hospital Anxiety and Depression Scale (HADS)	The nurse of the same gender, trained by the acupuncturist, provided reflexology massage. ❖ Foot reflexology massage.	Postintervention Anxiety levels Exp. group: 9.35±1.87 Cont. group: 10.25±2.11
Shafiei & Akbari (2017)/Iran	RCT	Isfahan Chamran Hospital	*N*= 72 patients who were underwent CABG • Exp. group: *n*=36 • Cont. group: *n*=36	The mood changes were measured using the Profile of Mood State questionnaire	A soothing baby oil that did not have any treatment value used on the participants’ left feet for one minute, followed by foot reflexology massage on the sole of the foot for 20 min. ❖ Foot reflexology massage.	Postintervention Anxiety levels Exp. group: 11.1±4.6 Cont. group: 21.4±6.1
Uzun Şahi̇n & Çi̇lingir (2022)/Turkey	RCT	Cardiovascular surgery clinic of a university hospital	*N*=70 patients • Exp. group: *n*=35 • Cont. group: *n*=35	STAI	On the second postoperative day, patients in the test group were administered a 20-min session of foot reflexology therapy was administered, 10 min for each patient’s foot ❖ Foot reflexology intervention	Postintervention Anxiety levels Exp. group: 38.0 Cont. group: 41.0 *p*=.028

*Note.* CABG=coronary artery bypass graft.

### Assessment of the Methodological Quality of Studies

The Joanna Briggs Institute (JBI) Critical Appraisal Checklists for RCTs were employed to assess the methodological quality of the studies included in this systematic review and meta-analysis (Barker et al., 2023). These checklists contain 13 questions for experimental research, each answered with one of four responses: yes, no, uncertain, and not applicable. The methodological quality rating of the included studies was deemed “mediocre” if fewer than 50% of the criteria were marked as “yes,” “moderate quality” if 51%–80% of the criteria were marked as “yes,” and “good quality” if over 80% of the criteria were marked as “yes” (Table 2).

**Table 2:** Critical Appraisal of Studies

Study	Questions of JBI Critical Appraisal Checklist for Randomized Controlled Studies														
	Q1	Q2	Q3	Q4	Q5	Q6	Q7	Q8	Q9	Q10	Q11	Q12	Q13	Total “Y”	Quality Score (%)
Abbaszadeh et al. (2018)/Iran	Y	U	Y	U	U	U	Y	Y	Y	Y	Y	Y	Y	9	Medium (69.2)
Bagheri-Nesami et al. (2014)/Iran	Y	Y	Y	Y	N	N	Y	Y	Y	Y	Y	Y	Y	11	Good (84.6)
Göktuna et al. (2023)/Turkey	Y	Y	Y	Y	N	Y	Y	Y	Y	Y	Y	Y	Y	12	Good (92.3)
Gunes et al. (2024)/Turkey	Y	Y	Y	Y	N	Y	Y	Y	Y	Y	Y	Y	Y	12	Good (92.3)
(Gunnarsdottir & Jonsdottir, 2007)/Iceland	Y	U	Y	U	U	U	Y	Y	Y	Y	Y	Y	Y	9	Medium (69.2)
Kolbadinejad et al. (2023)/Ian	Y	Y	Y	Y	N	Y	Y	Y	Y	Y	Y	Y	Y	12	Good (92.3)
(Shafiei, Z. 2017)/Iran	Y	Y	Y	N	N	Y	Y	Y	Y	Y	Y	Y	Y	11	Good (84.6)
Uzun Şahi̇n & Çi̇lingir (2022)/Turkey	U	U	Y	U	U	U	Y	Y	Y	Y	Y	Y	Y	8	Medium (61.5)
Article total quality score (%)	85.7	71.4	100	57.1	0	57.1	100	100	100	100	100	100	100		

*Note.* Q=question; Y=yes; N=no; NA=not applicable; U=unclear.

### Data Synthesis

Comprehensive Meta-Analysis Version 2 (CMA Ver. 2.0) was employed to evaluate effect sizes and conduct heterogeneity assessments in this study. Hedge’s *g* was utilized as the measure of effect size due to the significant variability in sample sizes across studies (Schmid et al., 2009), with the effect size categorized as small (0.2), medium (0.5), large (0.8), or very large (1.2; Hedges & Olkin, 2014). A *p* value of <.05 or a 95% CI was considered significant.

Heterogeneity among the included studies was evaluated using Cochran’s Q to evaluate the *I* ^2^ statistic, with *I* ^2^ values of 0%–40% indicating minimal to no heterogeneity, 30%–60% indicating moderate heterogeneity, 50%–90% indicating substantial heterogeneity, and 75%–100% indicating considerable heterogeneity (Higgins & Thomas, 2023). In this study, *I*² values of over 50% were used to indicate significant heterogeneity. Because the test statistics showed significant heterogeneity among the included studies (*I* ^2^ >75% and *p*<.05), a random-effects model was used to evaluate the level of anxiety with a 95% CI (Higgins & Thomas, 2023). All tests were analyzed using a two-tailed approach, with a *p*-value below .05 used to identify statistical significance (Higgins & Thomas, 2023). Funnel plot, Egger’s regression intercept, and Begg and Mazumder rank correlations were used to detect publication bias (Sedgwick, 2013). The statistical significance level was set at .05. A sensitivity analysis was conducted to assess the risk of bias in the study’s main outcome— the effect of reflexology on anxiety—considering factors such as the country where the study was conducted and the anxiety measurement tools used. This analysis focused on the country in which the study was conducted and the anxiety measurement tools used, as these factors may contribute to heterogeneity.

## Results

### Search Outcomes

**Figure 1:**
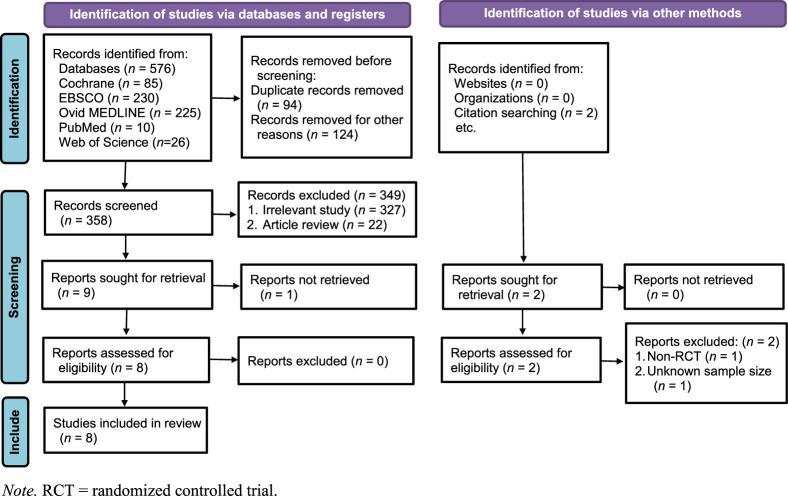
Flow Diagram of Preferred Reporting Items for Systematic Reviews and Meta-Analyses

Of the 576 articles obtained from the initial search, nine were retained for the full-text review after eliminating duplicate articles based on title and those that did not fulfill the search criteria based on a reading of the abstracts. Two researchers read the eight (*n*=8) studies eligible for analysis and included them in the meta-analysis based on meeting all the inclusion criteria. A manual review of the references of the included studies was performed and resulted in the preliminary identification of two additional studies. However, upon comprehensive review of these two studies, they were excluded from the meta-analysis for not meeting the inclusion criteria. A flow diagram explaining each step of the inclusion and exclusion process based on the PRISMA checklist is presented in Figure 1.

### Characteristics of the Included Studies and Participants

All of the studies included in the meta-analysis were randomized controlled trials (Abbaszadeh et al., 2018; Bagheri-Nesami et al., 2014; Göktuna et al., 2023; Gunes et al., 2024; Gunnarsdottir & Jonsdottir, 2007; Kolbadinejad et al., 2023; Shafiei & Akbari, 2017; Uzun Şahi̇n & Çi̇lingir, 2022). Countries where the studies were conducted included: Türkiye (Göktuna et al., 2023; Gunes et al., 2024; Uzun Şahi̇n & Çi̇lingir, 2022), Iran (Abbaszadeh et al., 2018; Bagheri-Nesami et al., 2014; Kolbadinejad et al., 2023; Shafiei & Akbari, 2017), and Iceland (Gunnarsdottir & Jonsdottir, 2007). All of the included studies were published between 2007 and 2024. Sample sizes ranged from 9 to 80 participants, and the total sample size of all studies was 485 (intervention group: 243; control group: 242). All of the participants had undergone open-heart surgery. In the eight studies included in the meta-analysis, anxiety levels were measured using three different scales. Six of these used the State-Trait Anxiety Inventory-STAI (Abbaszadeh et al., 2018; Bagheri-Nesami et al., 2014; Göktuna et al., 2023; Gunes et al., 2024; Gunnarsdottir & Jonsdottir, 2007; Uzun Şahi̇n & Çi̇lingir, 2022), one used the Hospital Anxiety and Depression Scale-HADS (Kolbadinejad et al., 2023), and one used the Profile of Mood State (POMS; Shafiei & Akbari, 2017).

The POMS, which is a standard questionnaire and its validity and reliability has been determined in various studies, including the research of Albert et al. (2009) and Babaee et al. (2012). The questionnaire contains 65 items in six dimensions: anxiety, depression, fatigue, confusion, anger, and ability. Each item is scored using a Likert scale with scores ranging from 0 (*never*) to 4 (*very much*).

The Hospital Anxiety and Depression Scale (HADS) consists of 14 items in two subscales: anxiety and depression. Each item is rated on a 4-point scale, and the maximum score for anxiety and depression is 21. Scoring 11 or above on both subscales is significant for psychological morbidity, with scores of 8–10 and 0–7 representing borderline and normal, respectively (McMordie, 1979).

The State-Trait Anxiety Inventory (STAI) consists of short statements used to determine state and trait anxiety levels separately. STAI is used to measure the level of anxiety in people over the age of 14. STAI consists of 40 items, with higher scores indicating higher anxiety levels (Spielberger et al., 1970). The details of the sample characteristics from the analyzed studies are outlined in Table 1.

### Characteristics of the Intervention

Foot reflexology was the most common intervention used (Abbaszadeh et al., 2018; Bagheri-Nesami et al., 2014; Gunes et al., 2024; Gunnarsdottir & Jonsdottir, 2007; Kolbadinejad et al., 2023; Shafiei & Akbari, 2017; Uzun Şahi̇n & Çi̇lingir, 2022). Hand reflexology was also applied in one study (Göktuna et al., 2023). None of the articles described the surgical methods used in open-heart surgery. Anxiety was measured using the STAI in eight studies, the HADS in one study, and the POMS questionnaire in one study. Reflexology areas targeted included the hand in one study and the foot in seven studies. Reflexology was applied after open-heart surgery in all of the studies. In this study, the anxiety levels reported by hospitalized patients undergoing open-heart surgery were based on the values ​​measured after the intervention (reflexology). In some of the studies, participants were unconscious on the first day after open-heart surgery, so anxiety could not be assessed. The duration of reflexology application in the studies included in the meta-analysis varied between 10 and 40 min (Table 1).

### Quality Assessment of Studies

The risk of bias assessment results for the included studies are presented in Table 2, with total quality scores ranging from 61.5% to 92.3%, indicating that a majority of the studies were of high quality. Randomization was well defined in seven studies and unclear in one study. Allocation to treatment groups was concealed in five studies and unclear in three studies. Treatment groups were similar in all studies. Participants were blinded to the intervention in four studies, unclear in three studies, and unblinded in one study. However, none of the studies blinded reflexology practitioners to the intervention. Outcome assessors were blinded in four studies, unclear in three, and unblinded in one study. In all of the studies included, the treatment groups were treated in the same way, follow-up was completed, participants were analyzed in the groups they were randomized to, results were measured in the same way and reliably, and results were analyzed using appropriate statistical methods and research designs.

### Outcomes of the Meta-Analysis

Eight studies investigating the effect of reflexology on anxiety in patients undergoing open-heart surgery were included in the meta-analysis. These studies included a total of 485 patients undergoing open-heart surgery. In the meta-analysis, common measurement times of anxiety were used in all studies. In all of the studies analyzed, anxiety levels were measured after a reflexology massage given after open-heart surgery.

The heterogeneity of the eight studies included in the meta-analysis was tested, with high heterogeneity observed (*Q* = 120.17, *df* = 7, *I*²=94.17, *Tau*²=1.39, *p*=.001). Thus, in light of this finding, systematic review and meta-analysis results should be interpreted with caution.

#### Effect of Reflexology on Anxiety After Open-Heart Surgery

**Figure 2:**
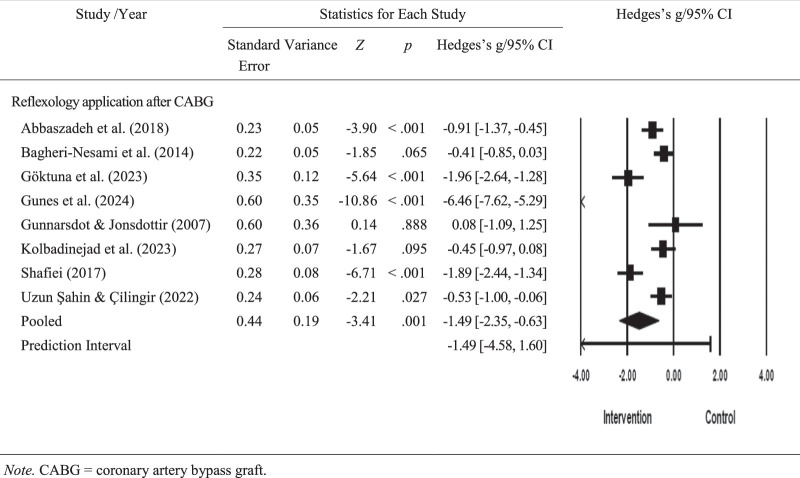
Forest Plot of Anxiety

The results of the pooled meta-analysis on the effect of reflexology on anxiety in patients undergoing open-heart surgery are shown in Figure 2. Based on the findings of studies covering 485 patients and the meta-analysis performed according to the random-effects model, it was found that reflexology significantly reduced anxiety (*g*=−1.49, 95% confidence interval [CI]=[−2.34, −0.63]; *z*=−3.40, *p*=.001; Figure 2).

According to the Egger test performed to detect publication bias, publication bias in the included studies was not statistically significant (intercept=−7.68, *SE*=4.19, 95% CI [−17.95, 2.57], *t*=1.83, *df*=6 and two-sided *p*>.05). The results of the Begg and Mazumdar correlation also showed publication bias to not reach statistical significance (*p*>.05). Duval and Tweedie’s (2000) trim and fill test was performed to identify possible missing studies and determine the effect of these studies on the main result of the meta-analysis. According to the results, the difference between the observed values ​​and the values ​​corrected to correct for the effect of publication bias was −0.44.

### Subgroup Analysis

Subgroup analyses were conducted based on the anxiety measurement tools used in the included studies and the countries where they were conducted. The significant effect remained in those studies that used the STAI (*g*=−1.62, 95% CI=[−2.77, −0.48]; *z*=−2.78, *p*=.005). However, in the subgroup analysis conducted on those using other scales, no significant effect was detected (*g*=−1.16, 95% CI [−2.57, 0.24]; *z*=−1.61, *p*=.10).

In the subgroup analysis conducted based on the countries where the studies were conducted, the significant effect continued in those conducted in Iran, which is consistent with the main finding of the meta-analysis (*g*=−0.90, 95% CI=[−1.53, −0.27]; *z*=−2.81, *p*=.005). Based on the combined results of those studies conducted in Turkey, the significant effect also continued, which is consistent with the main finding of the meta-analysis (*g*=−2.92, 95% CI=[−5.65, −0.20]; *z*=−2.10, *p*=.035). Because only one of the studies was conducted in Iceland, it was not included in the cross-country subgroup analysis.

## Discussion

The main purpose of this systematic review and meta-analysis study was to evaluate the effect of postoperative reflexology massage on anxiety in patients undergoing open-heart surgery. Some evidence of an effect was found in the reviewed literature. One was a review and meta-analysis in the literature on the effect of reflexology on anxiety in patients undergoing cardiovascular treatment (Chandrababu et al., 2019) that considered all cardiovascular surgical procedures, including minor and major invasive procedures. However, open-heart surgery, which was the main inclusion criterion for this study, is a larger cardiac procedure that requires a deeper incision due to the opening of the sternum. Thus, evidence from previous studies may not be sufficiently discriminatory for patients undergoing open-heart surgery.

Many studies have reported that individuals who have undergone open-heart surgery experience anxiety (Abbaszadeh et al., 2018; Bagheri-Nesami et al., 2014; Göktuna et al., 2023; Gunes et al., 2024). Anxiety activates the sympathetic nervous system, causing the release of epinephrine and norepinephrine. This response increases blood pressure, respiratory rate, and heart rate. Myocardial oxygen demand increases the risk of ischemia and dysrhythmia by causing cardiac workload (Göktuna et al., 2023; Mobini-Bidgoli et al., 2017). Evidence in the literature generally supports that reflexology has a positive effect on anxiety in individuals undergoing cardiovascular intervention (Abbaszadeh et al., 2018; Göktuna et al., 2023; Mobini-Bidgoli et al., 2017).

The positive findings in this study regarding the effect of reflexology massage on postoperative anxiety in patients undergoing open-heart surgery are consistent with the findings of a systematic review that reported reflexology reduces anxiety levels in patients undergoing different cardiovascular interventions (Chandrababu et al., 2019). Other studies in the literature have been conducted to evaluate the effects of nonpharmacological methods on anxiety in similar sample groups. In this context, the findings of this study are consistent with the findings of a systematic review, which found that the use of nonpharmacological methods after surgery can reduce anxiety in patients undergoing heart surgery (Soh et al., 2024). Other previous findings from different sample groups indicate that nonpharmacological methods can reduce anxiety (Staines et al., 2022; Zhang et al., 2021). Although the mechanism of action is yet to be fully explained, reflexology goes far beyond simple contact and pressure, reducing stress symptoms by causing physiological changes in the body. Reflexology may also improve mood by decreasing endorphin and enkephalin release (Chantrey, 2020; Göktuna et al., 2023; Mobini-Bidgoli et al., 2017).

Subgroup analyses were conducted based on the anxiety measurement tool used in each included study and on the country where each study was conducted. The combined results of the six studies using the STAI scale were significant and consistent with the main finding of this study. However, the combined results of those studies using the POMS and HADS scales were not significant. This discrepancy may be attributable to a lack of understanding of the scale or changes in the mood of patients during scale application (Hobbs et al., 2021). In the country-based subgroup analysis, the combined results of the four studies conducted in Iran and of the three studies conducted in Turkey were both significant and consistent with our main finding. The results from both countries show reflexology to have an effect on anxiety (Abbaszadeh et al., 2018; Bagheri-Nesami et al., 2014; Göktuna et al., 2023; Gunes et al., 2024; Kolbadinejad et al., 2023; Shafiei & Akbari, 2017; Uzun Şahi̇n & Çi̇lingir, 2022). The other country included in the meta-analysis (Iceland) was not included in the subgroup analysis because it was covered by only one study.

The findings of this meta-analysis provide preliminary evidence that reflexology interventions may have positive effects on patients undergoing open-heart surgery. From a nursing and health science perspective, these findings suggest reflexology may be considered a supportive practice in postcardiac intervention care (Abbaszadeh et al., 2018). However, these results should be interpreted with caution due to the low quality of the available evidence. Well-designed, high-quality randomized controlled trials are needed to provide stronger evidence of the efficacy and safety of reflexology (Wang et al., 2008; Whatley et al., 2022). This review is important to promote the integration of reflexology into behavioral and clinical health practices and contribute to comprehensive health care.

In conclusion, the current evidence and the findings of this study indicate that reflexology may help reduce anxiety levels in patients undergoing open-heart surgery in the postoperative period. However, due to the limited evidence in this area, the results of this meta-analysis should be interpreted with caution. Additional research will be needed to evaluate the effectiveness of interventions after open-heart surgery to generate further relevant evidence.

### Strengths and Limitations

The advantages of this research include the extensive search resources utilized, as well as the recency and good quality of most of the included studies. Also, the relatively large combined sample size in the meta-analysis is a notable strength that enhances the validity of the findings. This study is the first systematic review and meta-analysis designed to examine the effect of reflexology on anxiety in patients undergoing open-heart surgery. The results will help increase the scholarly understanding of the effect of nonpharmacological methods of treatment, such as reflexology, on anxiety. The findings of this meta-analysis provide evidence in support of reflexology offering beneficial effects to patients undergoing open-heart surgery, offering preliminary backing to health care providers and nurses incorporating reflexology treatments into interventions for individuals undergoing related procedures.

This study must be interpreted in light of several limitations. The data collected were self-reported by participants, which increases the risk of recall bias and thus may not accurately reflect the actual practices of the participants. Also, the different scales used in the studies included in the meta-analysis increase the difficulty of drawing definitive conclusions. Moreover, the inclusion of studies published in the English language only introduces the potential of language bias. In addition, the duration and frequency of reflexology interventions in the included studies were highly variable. Finally, the considerable heterogeneity among the included studies may reduce the strength of the findings. However, to address this issue, a random-effects model was used in the meta-analysis.

### Conclusions and Recommendations

The effectiveness of reflexology in reducing anxiety in patients undergoing open-heart surgery was evaluated in this meta-review analysis. The limitations of this current effort provide opportunities for future research. The findings suggest reflexology to be a safe and noninvasive bedside treatment for anxiety reduction in patients undergoing open-heart surgery during their time in the intensive care unit, as well as afterward. In summary, reflexology may be adopted by nurses and health care providers to complement traditional patient care, targeting the alleviation of anxiety. However, given the high heterogeneity, clinical and nonclinical effects of reflexology, and limitations in this study, the findings should be interpreted with caution. To advance knowledge and validate current findings in this area, further research is recommended.
